# Benign Mature Teratoma in Anterior Mediastinum

**DOI:** 10.14740/jocmr2270w

**Published:** 2015-07-24

**Authors:** Tae-Hoon No, Sang-Hoon Seol, Guang-Won Seo, Doo-Il Kim, Sung Yeun Yang, Chul Hoi Jeong, Youn-Ho Hwang, Ji Yeon Kim

**Affiliations:** a Department of Internal Medicine, Haeundae Paik Hospital, Inje University College of Medicine, Busan, Korea; b Department of Obstetrics and Gynecology, Haeundae Paik Hospital, Inje University College of Medicine, Busan, Korea; c Division of Thoracic Surgery, Haeundae Paik Hospital, Inje University College of Medicine, Busan, Korea; d Department of Pathology, Haeundae Paik Hospital, Inje University College of Medicine, Busan, Korea

**Keywords:** Mediastinum, Benign, Mature, Teratoma

## Abstract

Teratoma of mediastinum is rare germ cell tumor. Anterior mediastinum is the most common extragonadal site. Benign mediastinal teratoma accounts for 60% of all mediastinal germ cell tumors. Benign mature teratoma has excellent prognosis after surgical excision. We present a case of 20-year-old woman diagnosed as benign mature teratoma which compressed main pulmonary trunk. The patient underwent surgical excision.

## Introduction

A mediastinal teratoma is classified a tumor that derives from germ cells. The anterior mediastinum is the most frequent area of extragonadal germ cell tumors [1]. It is rare and usually benign. Most of the symptoms are as a result of compression of adjacent structures. Echocardiography shows usually extra-pericardial mass compressing major vascular structures. Chest CT is useful in the evaluation of location and its relationship to nearby structures. Complete surgical excision is treatment of choice for mediastinal teratoma.

## Case Report

**Figure 1:**
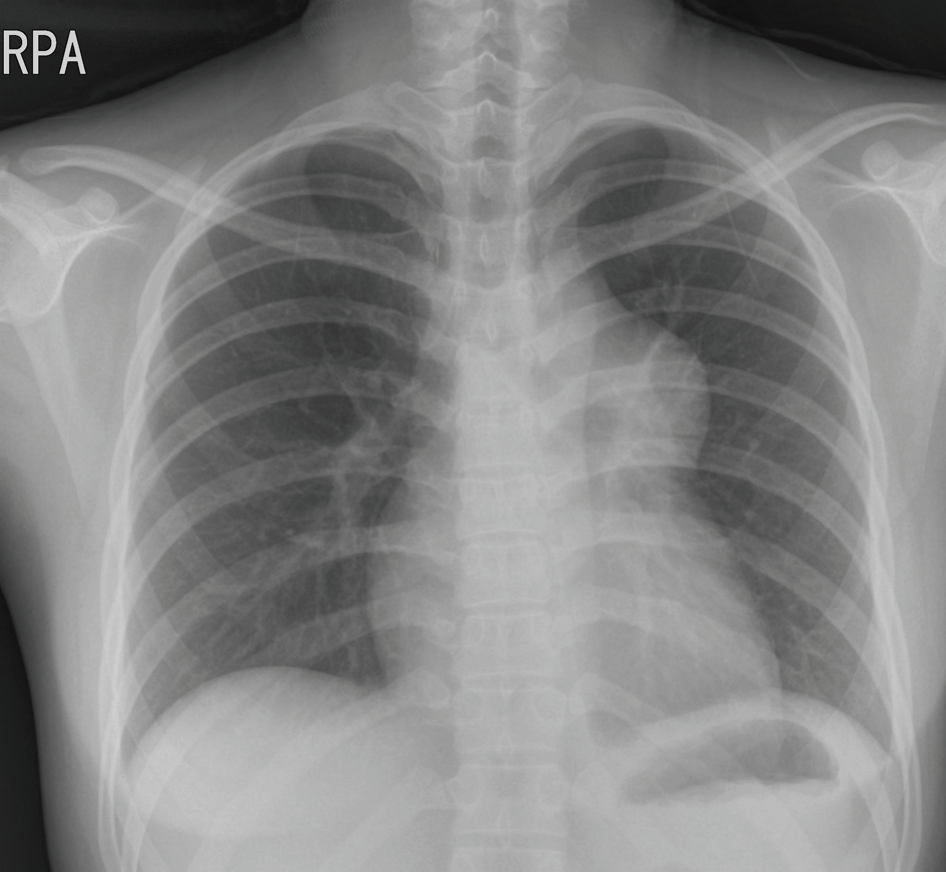
Chest X-ray showing protruding well-defined mass shadow in the left upper hilar space.

**Figure 2:**
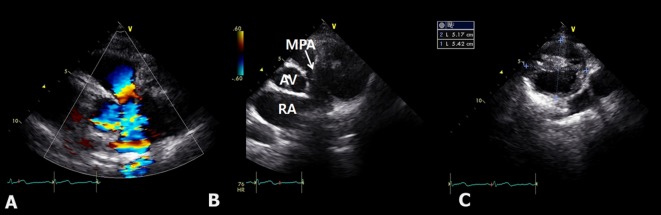
Transthoracic echocardiography demonstrating a turbulent color Doppler signal in the pulmonary trunk. (A) Compressing mass in the pulmonary trunk in the parasternal short axis view (B) multilocular cystic mass (C). RA: right atrium; MPA: main pulmonary artery; AV: aortic valve.

**Figure 3:**
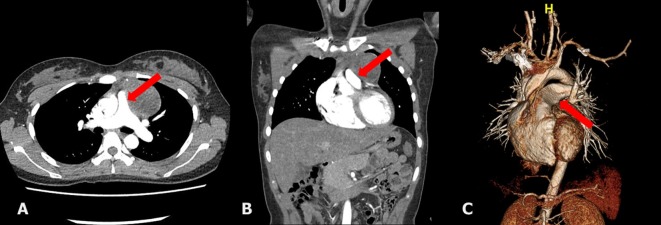
The chest CT showing a lobulated, inhomogeneous mass containing fat component in the mediastinum, compressing main pulmonary trunk (arrow), and no definite invasion to lung parenchyme (A, B, C).

**Figure 4:**
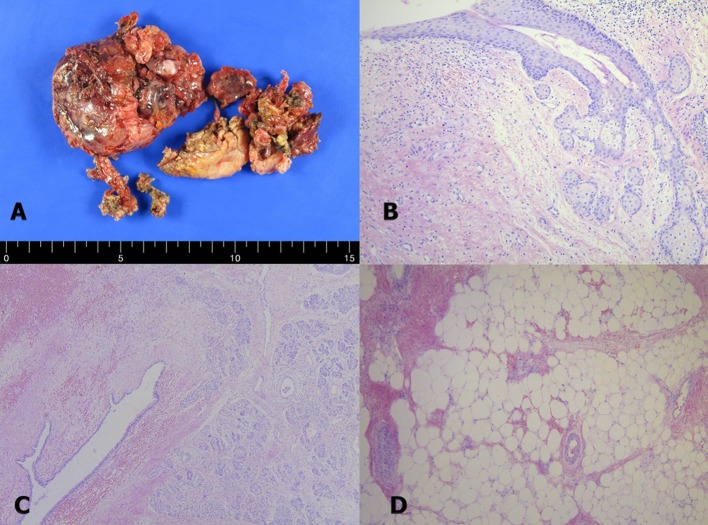
The pathologic findings. The excised specimen consisted of several fragments of heterogeneous soft tissue with disrupted outer surface (A). Photomicrographs of the mediastinal mass showed multiple areas of mature epidermis with sebaceous glands (B). In some areas of the mass, nests of respiratory epithelium and pancreatic tissue were observed (C). Foci of mature adipose tissue were also present in the tumor (D).

A 20-year-old woman who presented with increasing dyspnea on exertion was referred to our hospital for evaluation of a mediastinal mass. There were no complaints of fever or weight loss. On physical examination, electrocardiogram was normal. Chest X-ray revealed bulging mass opacity in the left upper hilar space (Fig. 1). Transthoracic echocardiography showed well-defined lobulated round mass compressing main pulmonary trunk in the parasternal short axis view (Fig. 2, Supplementary Videos 1, 2, www.jocmr.org). Chest CT demonstrated a well-demarcated lobulated mass of a 7 × 4 × 4 cm size with heterogeneous enhancement and fat component in the anterior mediastinum. The mass compressed main pulmonary trunk, and there was no definite invasion to lung (Fig. 3). The patient underwent surgical excision. Histopathologic examination confirmed the diagnosis of a benign mature teratoma (Fig. 4). The excised mass consisted of multiple areas of mature epidermis with sebaceous glands, nests of respiratory epithelium and pancreatic tissue, and foci of mature adipose tissue. The patient was uneventful and discharged without complication.

## Discussion

A teratoma is classified a tumor that derives from germ cells. It is composed of well-differentiated tissues derived from more than one of the all three embryonic cell layers: ectoderm, mesoderm and endoderm [2]. It is usually found both in gonadal organs and at extragonadal sites such as mediastinum, pineal area, sacrococcygeum. If the tumor is comprised of well-differentiated elements, then the tumor is referred to as a mature teratoma. Mature teratoma of the mediastinum is generally benign, although they have malignant potential [3, 4]. Benign mediastinal teratoma is seen in equal frequency in women and men, but malignant teratoma is more common in men [5, 6]. Mature teratoma of the mediastinum usually grows slowly. The patients are often asymptomatic, and the tumor is found incidentally on chest X-ray [7]. Most of symptoms are related to compression of nearby structures such as chest pain, dyspnea, cough or pulmonary infection. Chest X-ray often shows an anterior mediastinal mass. Chest CT is usually the imaging modality of choice for the diagnosis of mediastinal teratoma [8]. It shows the location and extent of adjacent structures of the tumors as well as intrinsic elements including soft tissue, fat component, and calcification [8]. The presence of a fat-fluid level is considered specific for the diagnosis of teratoma but is seldom seen radiographically [9]. Echocardiography shows usually extra-pericardial mass and compressing major vascular structures. MRI is also utilized for evaluation of mediastinal teratoma. MRI reveals mass of heterogeneous signal intensity, is sensitive in depicting infiltration of the adjacent structures by fat plane obliteration, and is performed as an ancillary study [10]. Complete surgical excision is the treatment of choice for mediastinal teratoma because of probable complications such as compression of adjacent structures, rupture or malignant transformation.

### Conclusion

Mediastinal mature teratoma is uncommon. This slow growing tumor is usually asymptomatic and is often detected incidentally on chest X-ray. Echocardiography and chest CT is helpful to evaluate compression of adjacent structures. Complete surgical resection should be performed for a benign teratoma and prognosis is excellent.
